# Supplementary material for: A Novel Tongue Squamous Cell Carcinoma Cell Line Escapes from Immune Recognition due to Genetic Alterations in HLA Class I Complex
Source: Cells. 2022 Dec 22;12(1):35. doi: 10.3390/cells12010035 (PMC9818362; doi:10.3390/cells12010035)
Supplement: Supplementary file 1 [file cells-12-00035-s001.zip › Supplementary Figures.pdf]

Supplementary Material

Supplementary Figures

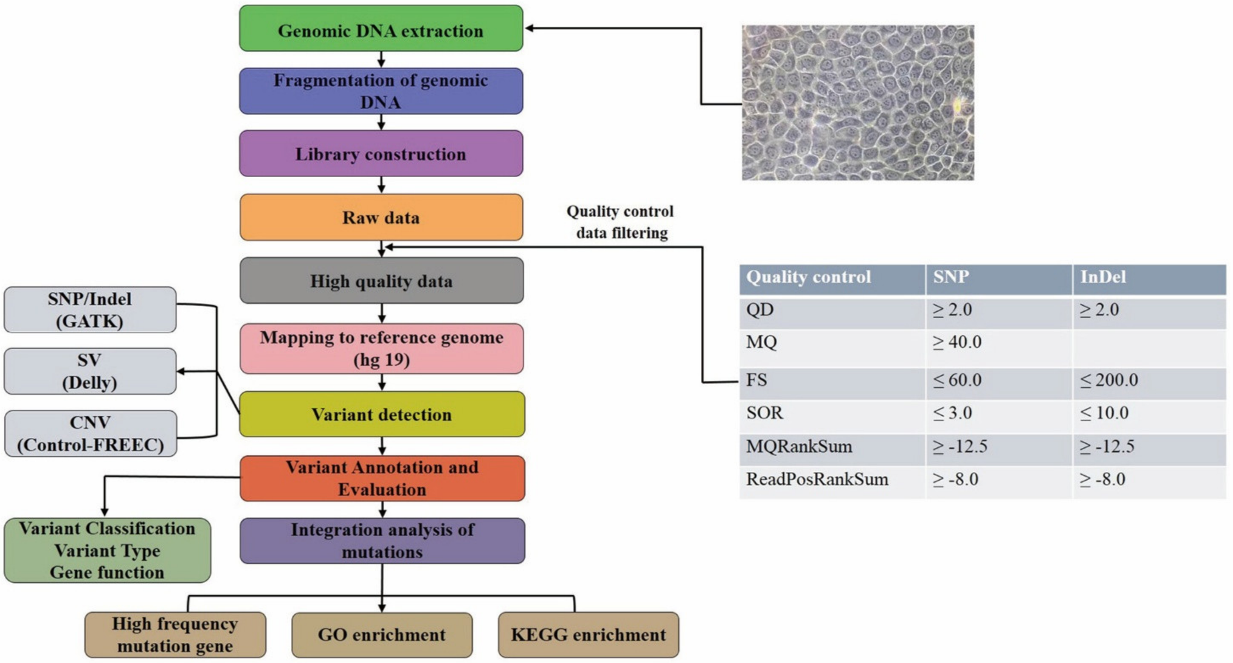

Figure S1. Flow chart of WES.

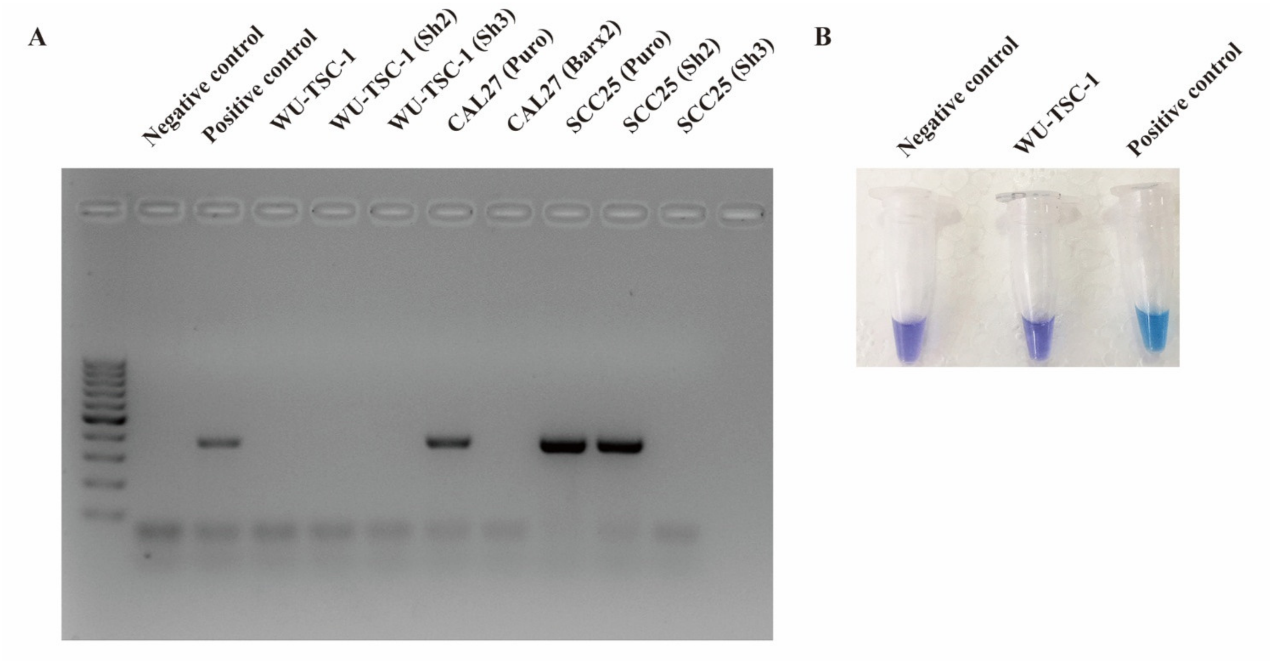

Figure S2. Detection of mycoplasma contamination in WU-TSC-1

(A) Detection of mycoplasma contamination in WU-TSC-1 cells by PCR. Product size:

350bp

F1: A(A/C)ACCATGGGAG(C/T)TGGTAAT

R1: TTCATCGACTTTCAGACCCAAGGCAT

(B) Detection of mycoplasma contamination in WU-TSC-1 cells by MycoBlue Mycoplasma detector kit.

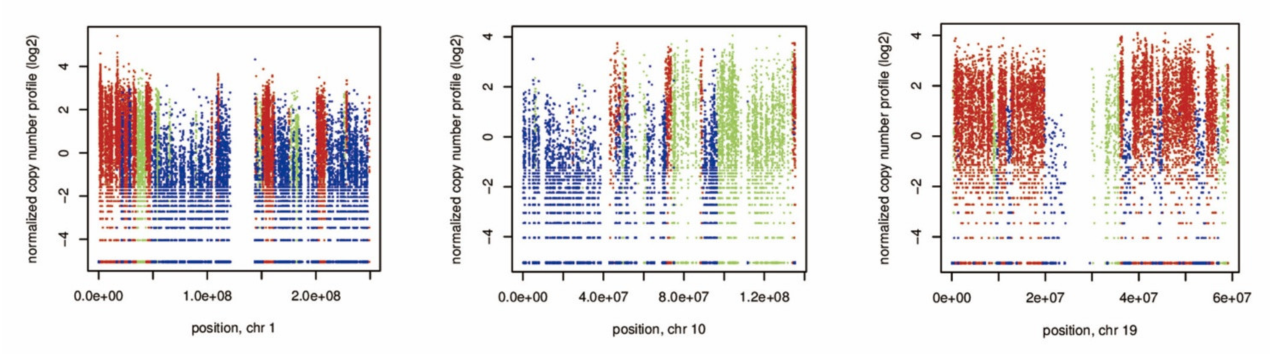

Figure S3. Duplications of CNV were mainly located on chromosome 1, 10 and 19
