# Supplementary material for: A Novel Tongue Squamous Cell Carcinoma Cell Line Escapes from Immune Recognition due to Genetic Alterations in HLA Class I Complex
Source: Cells. 2022 Dec 22;12(1):35. doi: 10.3390/cells12010035 (PMC9818362; doi:10.3390/cells12010035)
Supplement: Supplementary file 1 [file cells-12-00035-s001.zip › Supplementary Methods.pdf]

″

Detailed Protocol for Primary TSCC Cell Culture

1. Tissue dissociation

   Tongue SCC tissue was cut into small pieces (about 1 mm^3) and digested with 1mg/ml type IV collagenase (Sigma-Aldrich, USA), 0.2mg/ml DNase I enzyme (BioFroxx, Germany) and 0.1mg/ml hyaluronidase (Sigma-Aldrich, USA) for 1 h at 37 °C to ensure the tissue pieces fully dissociated.

2. Primary culturing

   Both digested tissue blocks and filtered supernatants were transferred for primary culture. Growth medium was consisted of DMEM/High glucose supplemented with 20% fetal bovine se-rum (Hyclone, USA), 200 U/ml penicillin, 200 μg/ml streptomycin, and 600ng/ml Hydro-cortisone in a CO2 incubator (5% CO2 and 95% air) at 37 °C. Growth medium was changed every 2-3 days.

3. Cancer cells purification

   For purifying cancer cells, differential trypsinization method was used given the different sensitivity to trypsin between stromal cells and epithelial cells. Cells were treated with a solution of 0.25% trypsin for 2 min. Fibroblasts, which are more sensitive than epithelial cells to trypsin, detached first, leaving behind epithelial cells. Digestion time ensures removal of spindle-shaped fibroblasts without affecting cancer cells. The previous trypsin-digestion step was repeated at every cell passage until the remaining cells were all cancer cells.
