# Supplementary material for: A Novel Tongue Squamous Cell Carcinoma Cell Line Escapes from Immune Recognition due to Genetic Alterations in HLA Class I Complex
Source: Cells. 2022 Dec 22;12(1):35. doi: 10.3390/cells12010035 (PMC9818362; doi:10.3390/cells12010035)
Supplement: Supplementary file 1 [file cells-12-00035-s001.zip › Table S1.pdf]

Table S1: Detailed clinical characterization of the donor patient.

Diagnosis Age	42
Gender	Female
Alcohol History Documented	No
Smoking History Documented	No
Areca nut chewing Documented	No
Race Category	Asian
Prior Cancer Diagnosis Occurrence	No
Tumor Site	Right side of base of tongue
Year Cancer Initial Diagnosis	2020
Neoadjuvant Therapy Type Administered Prior To Resection	No
Did patient start adjuvant postoperative radiotherapy?	No
Surgical Manner	Enlarged resection of the base of the tongue lesions+ Partial mandibulectomy + Skin flap graft+ Radical neck dissection
Disease Surgical Margin Status	Negative
Neoplasm Histologic Type Name	Squamous Cell Carcinoma
Neoplasm AJCC Primary Tumor T Stage	T3
Neoplasm AJCC Regional Lymph Node N Stage	N0
Neoplasm AJCC Distant Metastasis M Stage	M0
Neoplasm Histologic Grade	GI-GII
Expression of PD-L1	Negative
Adjuvant Postoperative Pharmaceutical Therapy	No
Did patient start adjuvant postoperative radiotherapy?	No
Disease Free (Months)	4
Disease Free Status	Recurred/Progressed
